# Supplementary material for: Modeling glioblastoma heterogeneity as a dynamic network of cell states
Source: Mol Syst Biol. 2021 Sep 16;17(9):e10105. doi: 10.15252/msb.202010105 (PMC8444284; doi:10.15252/msb.202010105)
Supplement: Supplementary file 5 — Source Data for Figure 3 [file MSB-17-e10105-s001.zip › Figure3A_sourcedata/GSEA_3065/nosigdb_state1.GseaPreranked.1622718409535/SUVA_G2M_PMID_31327527.html]

Details for gene set SUVA\_G2M\_PMID\_31327527[GSEA]

|  || Dataset | state1 |
| Phenotype | NoPhenotypeAvailable |
| Upregulated in class | na\_pos |
| GeneSet | SUVA\_G2M\_PMID\_31327527 |
| Enrichment Score (ES) | 0.599274 |
| Normalized Enrichment Score (NES) | 1.8685659 |
| Nominal p-value | 0.0 |
| FDR q-value | 0.0010472586 |
| FWER p-Value | 0.021 |
Table: GSEA Results Summary

  

Fig 1: Enrichment plot: SUVA\_G2M\_PMID\_31327527      
 Profile of the Running ES Score & Positions of GeneSet Members on the Rank Ordered List

  

| PROBE | GENE SYMBOL | GENE\_TITLE | RANK IN GENE LIST | RANK METRIC SCORE | RUNNING ES | CORE ENRICHMENT || 1 | TUBA1C |  |  | 24 | 0.619 | 0.1386 | Yes |
| 2 | TUBB4B |  |  | 67 | 0.429 | 0.2323 | Yes |
| 3 | H2AFZ |  |  | 153 | 0.321 | 0.2967 | Yes |
| 4 | MZT1 |  |  | 449 | 0.216 | 0.3159 | Yes |
| 5 | CDC20 |  |  | 469 | 0.212 | 0.3623 | Yes |
| 6 | AURKA |  |  | 550 | 0.196 | 0.3987 | Yes |
| 7 | CCNB1 |  |  | 583 | 0.189 | 0.4385 | Yes |
| 8 | HMGB3 |  |  | 907 | 0.144 | 0.4384 | Yes |
| 9 | CDCA3 |  |  | 994 | 0.134 | 0.4603 | Yes |
| 10 | TROAP |  |  | 1134 | 0.119 | 0.4733 | Yes |
| 11 | CCNB2 |  |  | 1232 | 0.110 | 0.4884 | Yes |
| 12 | KIF4A |  |  | 1303 | 0.103 | 0.5047 | Yes |
| 13 | CKAP2L |  |  | 1359 | 0.099 | 0.5216 | Yes |
| 14 | PLK1 |  |  | 1403 | 0.095 | 0.5389 | Yes |
| 15 | RANGAP1 |  |  | 1414 | 0.094 | 0.5593 | Yes |
| 16 | ANP32E |  |  | 1706 | 0.075 | 0.5468 | Yes |
| 17 | CCNA2 |  |  | 1835 | 0.068 | 0.5493 | Yes |
| 18 | TACC3 |  |  | 1949 | 0.063 | 0.5521 | Yes |
| 19 | RACGAP1 |  |  | 1994 | 0.060 | 0.5613 | Yes |
| 20 | NCAPD2 |  |  | 2121 | 0.054 | 0.5608 | Yes |
| 21 | ECT2 |  |  | 2150 | 0.053 | 0.5700 | Yes |
| 22 | CDCA8 |  |  | 2166 | 0.052 | 0.5804 | Yes |
| 23 | KIF2C |  |  | 2224 | 0.050 | 0.5861 | Yes |
| 24 | SMC4 |  |  | 2308 | 0.047 | 0.5883 | Yes |
| 25 | CDC25B |  |  | 2370 | 0.044 | 0.5922 | Yes |
| 26 | GPSM2 |  |  | 2477 | 0.040 | 0.5906 | Yes |
| 27 | KNSTRN |  |  | 2515 | 0.039 | 0.5958 | Yes |
| 28 | SPAG5 |  |  | 2566 | 0.038 | 0.5993 | Yes |
| 29 | CKAP2 |  |  | 2763 | 0.031 | 0.5864 | No |
| 30 | ARHGAP11A |  |  | 2814 | 0.030 | 0.5882 | No |
| 31 | BUB1 |  |  | 2888 | 0.028 | 0.5872 | No |
| 32 | KIF20A |  |  | 2897 | 0.028 | 0.5926 | No |
| 33 | TMPO |  |  | 3093 | 0.023 | 0.5780 | No |
| 34 | FOXM1 |  |  | 3117 | 0.022 | 0.5807 | No |
| 35 | OIP5 |  |  | 3357 | 0.017 | 0.5602 | No |
| 36 | TTK |  |  | 3724 | 0.009 | 0.5252 | No |
| 37 | REEP4 |  |  | 4020 | 0.004 | 0.4961 | No |
| 38 | KIFC1 |  |  | 5017 | -0.013 | 0.3977 | No |
| 39 | MXD3 |  |  | 5096 | -0.014 | 0.3930 | No |
| 40 | BUB1B |  |  | 5187 | -0.015 | 0.3873 | No |
| 41 | KPNA2 |  |  | 5461 | -0.020 | 0.3640 | No |
| 42 | KIF11 |  |  | 6477 | -0.038 | 0.2695 | No |
| 43 | CENPF |  |  | 6902 | -0.047 | 0.2372 | No |
| 44 | PSRC1 |  |  | 8973 | -0.141 | 0.0588 | No |
| 45 | ARL6IP1 |  |  | 9038 | -0.146 | 0.0855 | No |
Table: GSEA details [plain text format]

  

Fig 2: SUVA\_G2M\_PMID\_31327527: Random ES distribution      
 Gene set null distribution of ES for **SUVA\_G2M\_PMID\_31327527**
